# Cholera outbreak caused by drinking contaminated water from a lakeshore water-collection site, Kasese District, south-western Uganda, June-July 2015

**DOI:** 10.1371/journal.pone.0198431

**Published:** 2018-06-27

**Authors:** Gerald Pande, Benon Kwesiga, Godfrey Bwire, Peter Kalyebi, AlexArio Riolexus, Joseph K. B. Matovu, Fredrick Makumbi, Shaban Mugerwa, Joshua Musinguzi, Rhoda K. Wanyenze, Bao-Ping Zhu

**Affiliations:** 1 Uganda Public Health Fellowship Program – Field Epidemiology Track, Kampala, Uganda; 2 Ministry of Health Uganda, Kampala, Uganda; 3 Makerere University School of Public Health, Kampala, Uganda; 4 United States Centers for Disease Control and Prevention, Kampala, Uganda; 5 Divison of Global Health Protection, Center for Global Health, United States Centers for Disease Control and Prevention, Atlanta, Georgia, United States of America; Australian National University, AUSTRALIA

## Abstract

On 20 June 2015, a cholera outbreak affecting more than 30 people was reported in a fishing village, Katwe, in Kasese District, south-western Uganda. We investigated this outbreak to identify the mode of transmission and to recommend control measures. We defined a suspected case as onset of acute watery diarrhoea between 1 June and 15 July 2015 in a resident of Katwe village; a confirmed case was a suspected case with *Vibrio cholerae* cultured from stool. For case finding, we reviewed medical records and actively searched for cases in the community. In a case-control investigation we compared exposure histories of 32 suspected case-persons and 128 age-matched controls. We also conducted an environmental assessment on how the exposures had occurred. We found 61 suspected cases (attack rate = 4.9/1000) during this outbreak, of which eight were confirmed. The primary case-person had onset on 16 June; afterwards cases sharply increased, peaked on 19 June, and rapidly declined afterwards. After 22 June, eight scattered cases occurred. The case-control investigation showed that 97% (31/32) of cases and 62% (79/128) of controls usually collected water from inside a water-collection site “X” (OR_M-H_ = 16; 95% CI = 2.4–107). The primary case-person who developed symptoms while fishing, reportedly came ashore in the early morning hours on 17 June, and defecated “near” water-collection site X. We concluded that this cholera outbreak was caused by drinking lake water collected from inside the lakeshore water-collection site X. At our recommendations, the village administration provided water chlorination tablets to the villagers, issued water boiling advisory to the villagers, rigorously disinfected all patients’ faeces and, three weeks later, fixed the tap-water system.

## Introduction

Cholera is a diarrhoeal disease caused by the bacterium *Vibrio cholerae*. Approximately 20% of those infected with *V*. *cholerae* develop acute, watery diarrhoea and 10–20% of those infected develop severe diarrhoea and vomiting [1]. The incubation period for cholera is short (a few hours–5 days for most subtypes) [2]. Cholera can spread quickly in places with poor water and sanitation conditions once the organism is introduced[3]. Outbreaks are usually caused by consumption of contaminated water or food [2, 3]. Since cholera has a relatively high infectious dose (10^4^ organisms [4]), it often requires heavy contamination of drinking water or multiplication of the pathogen in the contaminated food to cause outbreaks.

Since the 19^th^century, the world has experienced 7 cholera pandemics, which have caused millions of deaths. The current pandemic, 7^th^ overall, started in South Asia in 1961 and has spread to all World Health Organization (WHO) regions [3]. In 2016 alone, more than 132,000 cases of cholera were reported in 38 countries worldwide, including 2420 deaths, with a case fatality rate of 1.8% [5]. Of all continents, Africa has been the worst affected during the current pandemic—in 2016, 17 African countries reported 54% of all global cases. The case-fatality rate in Africa (2.5%) was also well above the global level (1.8%); of the 2420 reported deaths globally in 2016, 1762 (73%) occurred in Africa [5]. Inadequate water and sanitation conditions have been identified as the driving force for the cholera epidemic in Africa [6].

Cholera continues to be an important public health problem in Uganda. Outbreaks, sometimes prolonged or wide spread, have occurred since the disease was first reported in 1971[7]. Since 1997, cholera cases have been reported annually in Uganda, including a major epidemic that occurred in 1998, with nearly 50,000 reported cases [8]. Districts bordering the Democratic Republic of Congo (DRC), South Sudan and Kenya, as well as urban slums in Kampala were the most affected areas [8]. Despite the frequent occurrence of outbreaks in Uganda, during the past 10 years only two have been rigorously investigated[9, 10] following the standard steps in an outbreak investigation [11].

On 20 June 2015, a cholera outbreak, with more than 30 reported cases including 19 positive by a rapid diagnostic test (RDT) and eight culture-confirmed, occurred in Katwe Village, a fishing village in Kasese District, south-western Uganda. We conducted an investigation to assess the scope of the outbreak, identify the mode of transmission, and inform control and prevention measures.

## Methods

### Description of the area where the outbreak occurred

**Fig 1:**
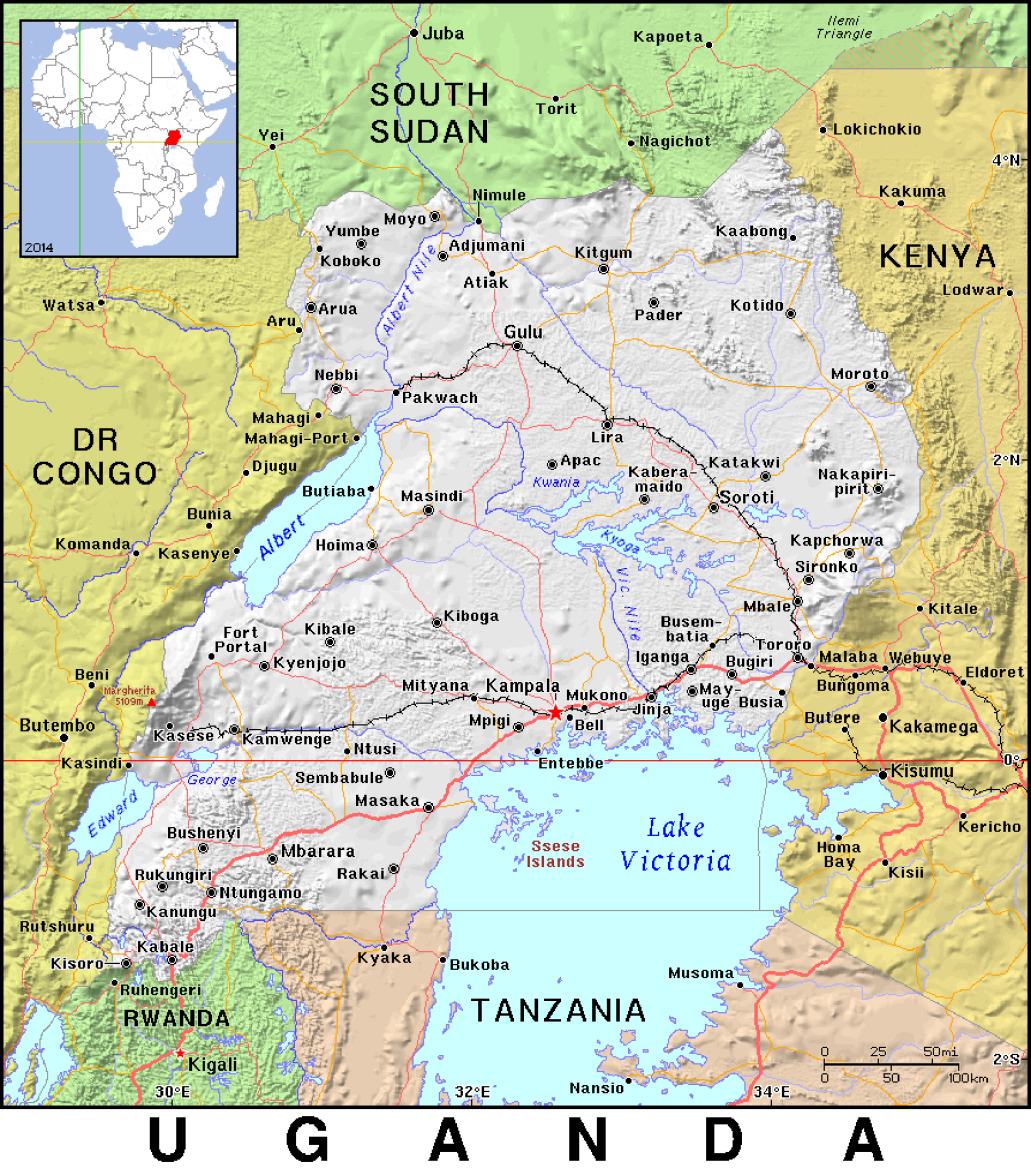
Location of Katwe Village in Uganda, where a cholera outbreak occurred during June–July 2015. (Map source: http://ian.macky.net/pat/map/ug/ug.html, public domain, accessed 20^th^ November 2017).

Kasese District is located in south-western Uganda, bordering DRC (Fig 1). Cholera outbreaks have been reported in the district before [12]. The district has a population of 12,324 persons according to the 2014 census. Katwe Village (0°08'44.5"S, 29°53'05.8"E) is located inside the Queen Elizabeth National Park between Lake Edward and Lake Katwe (Fig 1).

The village consists of five settlement zones, i.e. Kyakitale, Top Hill, Kyarukara, Kiganda, and Rwenjubu. The main economic activity in the village is fishing. The village has one health facility, the Katwe Health Centre III. After the outbreak occurred, the local government established a cholera treatment centre at this health centre.

### Case definition and identification

After reviewing the common signs and symptoms of the initial case-persons, we constructed a two-tiered case definition: A *suspected case* was onset of acute watery diarrhoea in a Katwe Village resident from 1 June to 15 July, 2015; a *confirmed case* was a suspected case with *V*. *cholerae* cultured from a stool specimen.

We reviewed patient records at the cholera treatment centre at Katwe Health Centre III between 1 June and 15 July, 2015 to identify patients that met the definition for suspected cases. We also reviewed the data in the Health Management Information System, an electronic disease reporting system managed by the Ministry of Health of Uganda. This data base contained basic information of patients with reportable conditions, including name, age, sex, residence, date of admission, date of hospitalization and clinical symptoms. To improve the completeness of case finding, we worked with health workers and key informants who were accurately aware of health-related issues in the community, including members of village health teams and community leaders, to identify diarrhoeal patients in the community that met the case definition. The community leaders also encouraged every one with diarrhoea to report to the nearest health facility. Persons who met the definition for suspected cases were identified and interviewed to collect data on their clinical presentations and potential exposures using a case-investigation questionnaire.

### Descriptive epidemiology and hypothesis generation

To generate the hypothesis on the mode of transmission for this outbreak, we evaluated the distribution of the case-patients’ clinical presentations, constructed an epidemic curve to describe patterns of case-persons’ dates of onset, and computed attack rates by age, sex, place of residence, and educational level. We also conducted hypothesis-generation interviews of 10 case-personsregarding potential exposures during the five days prior to their symptom onset, and community leaders on whether there had been any large gatherings that could explain this outbreak. Based on the descriptive epidemiology findings, we developed a hypothesis on the probable mode of transmission for this outbreak, and estimated the point in time when the exposure likely had occurred.

### Case-control investigation

To test the hypothesis formed from the descriptive epidemiology, we conducted a case-control investigation. Because the vast majority of cases occurred in the Kyarukara settlement zone, we recruited suspected case-patients in this zone to participate in the case-control investigation. If a household had more than one eligible case-person, only one was invited to participate.

Controls were selected among residents of Kyarukara settlement zone who had novomiting or diarrhoea from 1 June 2015 to the time of the investigation. To select controls randomly, we obtained a list of all households in Kyarukara settlement zone, and randomly selected four times as many control-households as the number of cases by using paper lots. For each case-patient, we identified among the randomly selected households four age-matched controls (i.e., within the same five-year age-group) whose residence was closest to that of the case-patient. If a household has diarrhoeal or vomiting patients since June 1, were placed it with one without such patients in the same neighbourhood.

We used a structured questionnaire to collect information from case-persons and controls on exposures related to food and drinking water exposures, including the usual source of drinking water, and the usual water-collection site the kind of food they usually ate, whether they usually ate hot or cold food. However, considering that the epidemic curve indicated a point-source exposure, and that community interviews revealed no large gatherings during which sharing of food might explain this outbreak, we presumed that food exposure was unlikely a major driver for this outbreak except for perhaps the sporadic cases after 22 June. Therefore, we only analysed water exposure. We also collected demographic data (e.g., age, sex, occupation, and education). We trained members of the village health team to administer the questionnaires.

To account for the matched design for the case-control investigation, we calculated the Mantel-Haenszel adjusted odds ratios (OR_M-H_) and their associated 95% confidence intervals (CI) [13].

### Laboratory and environmental investigations

We collected stool samples from case-patients and transported them in Cary-Blair media to the clinical laboratory at the Bwera Hospital for laboratory testing. The stool specimens were first tested using an RDT [14]. RDT-positive specimens were plated on agar for bacterial culture [15]. For environmental investigation, the team visited all settlements within the village, observed the water-collection sites used by the village residents, assessed the general water and sanitation conditions in the community, and interviewed the community leaders about water and sanitation practices.

## Ethical considerations

The Ministry of Health of Uganda gave the directive and approval to conduct this investigation. Additionally, the office of the Associate Director for Science, CDC/Uganda, determined that the primary purpose of this investigation was to verify, characterize and control an outbreak; hence it was not human subjects research. We obtained verbal informed consent from case-persons and controls above 18 years of age. For participants under18 years of age, we sought verbal consent from their parents or guardians and assent from respondents. We assuredthe case-patients and controls that their participation was completely voluntary and there would be no negative consequences should they decided not to participate.

## Results

### Descriptive epidemiology and hypothesis generation

**Fig 2:**
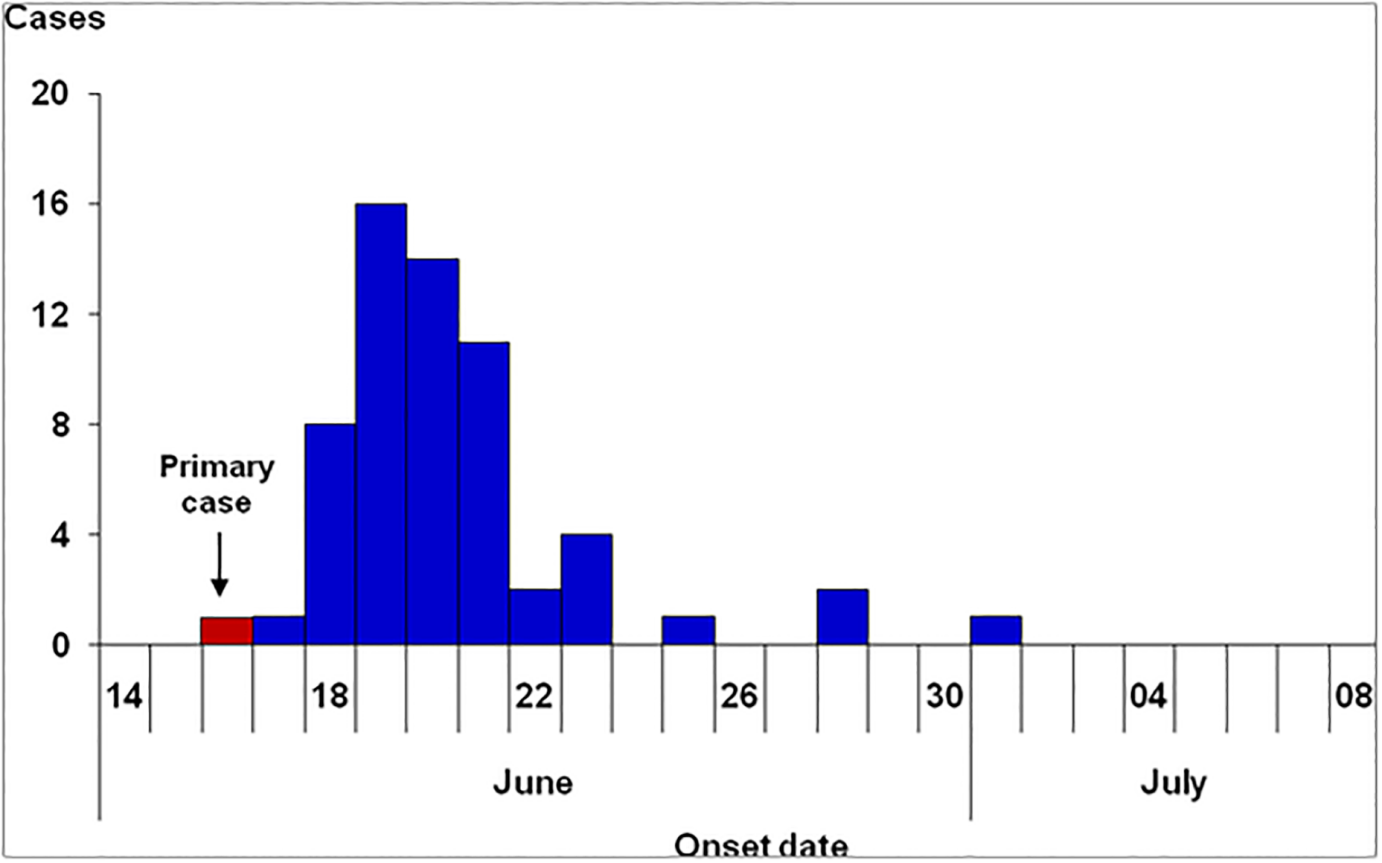
Onset of suspected cases of cholera during an outbreak in Katwe Village, south-western Uganda, June–July 2015. This epidemic curve indicated a point-source outbreak with exposure on 16 or 17 June, followed by community transmission after 22 June.

We identified 61 suspected cases in the village, with no deaths. Of these cases, 19 tested positive by RDT and eightwere culture-confirmed to be cholera 01 serotype Inaba. The clinical symptoms included acute watery diarrhoea (100%), vomiting (64%), abdominal pain (79%), and self-reported fever (2%). The primary case occurred on 16 June 2015. Cases increased sharply on 18 June, peaked on 19 June, and rapidly declined thereafter. After 22 June, eight additional cases occurred sporadically in the community; the last case occurred on 1 July. This epidemic curve indicated a point-source exposure pattern in the beginning phase of the outbreak[16], followed by occurrence of scattered cases in the community (Fig 2).

The pattern of the epidemic curve in relation to the incubation period for a particular disease can give clues on the source and time of exposure [16]. For cholera O1 serotype Inaba, the median incubation period is 3 days (range: a few hours to 5 days) [2]. Since this is a point-source outbreak, we determined the time of occurrence of the point-source exposure as follows [16]: Counting back 3 days (i.e., median incubation period) from 19 June (i.e., peak of the epidemic curve), we estimated that the exposure occurred on 16 June; similarly, counting back 5 days (i.e., maximum incubation period) from 22 June (i.e., end of the initial point-source epidemic curve), we estimated that the exposure occurred on 17 June. Therefore the exposure likely occurred between 16 and 17^th^ June 2015.

The descriptive epidemiologic analysis on the distribution of cases by place of residence showed that, of the five settlements, Kyarukara had the highest attack rate (21/1000), followed by Top Hill (0.67/1000) and Kiganda (0.36/1000); whereas Kyakitale and Rwenjubu had no cases. The analysis by demographic characteristics revealed that the attack rates were similar between male-residents (4.5/1000) and female-residents (5.5/1000), and among different age groups (Table 1). These data suggested that the exposure that caused this outbreak affected all socio demographic subgroups, and mainly affected residents of the Kyarukara settlement zone.

**Table 1:** Attack rate by settlement zone, sex, and age during a cholera outbreak in Katwe Village, south-western Uganda, June–July 2015.

Characteristics	Population	Cases	Attack Rate (/1000)
**Overall**	12324	61	4.9
**Settlement Zone**			
Rwenjubu	2396	0	0
Kyakitale	2813	0	0
Top Hill	1500	1	0.67
Kyarukara	2855	59	21
Kiganda	2760	1	0.36
**Sex**			
Male	6466	29	4.5
Female	5858	32	5.5
Age (years)*			
0–9	4157	23	5.5
10–19	3159	11	3.5
20–29	2025	14	6.9
30–39	1236	3	2.4
40–49	792	3	3.8
50–59	460	2	4.3
≥60	495	3	6.1

* Two persons were missing age information.

Interviews with community leaders revealed no large community gatherings on 16 and 17 June where contaminated food might have caused the initial point-source outbreak indicated by the epidemic curve. On the other hand, nine of the ten case-persons interviewed usually collected their drinking water from a lakeshore water-collection site “X” (described later), which served Kyarukara, the most affected settlement zone. Also, none of the 10 case-persons interviewed reported treating or boiling their drinking water.

In summary, the descriptive epidemiology analysis and the hypothesis-generating interviews clearly indicated that this was probably a waterborne outbreak caused by drinking contaminated water (likely from water-collection site X) between 16 and 17 June.

### Case-control investigation findings

In the case-control investigation, case-persons and controls did not differ significantly in the distributions of age (p = 0.33), sex (p = 0.75) and education level (p = 0.57). However, 97% of the 32 case-persons compared with 62% of the 128 controls usually collected their drinking water from water-collection site X (OR_M-H_ = 16, 95% CI: 2.4–107). On the other hand, individuals who did not treatment drinking water (i.e., boiling, filtering, or chlorination with a chlorine tablet) had less odds of being cholera cases (OR_M-H_ = 0.29, 95% CI: 0.099–0.82) (Table 2).

**Table 2:** Exposures significantly associated with cholera infection, KatweVillage, south-western Uganda, June–July 2015.

Exposure factors	% cases (n = 32)	% controls (n = 128)	OR_M-H_	95%CI
Water-collection site usually used				
Site X*	97	62	16	2.4–107
All other sites	3.0	38		
Any treatment of drinking water^#^				
No	9.4	33	0.29	0.099–0.82
Yes	11	67		

*An area on the lakeshore which was protected by rocks and a fence to protect water collectors from attacks by lake animals (such as crocodiles and hippopotamuses);

^#^Boiling, filtering, or chlorination with chlorine tablets.

### Findings from the interview of the primary case-person

The primary case-person was a fisherman. During an in-depth interview, he described that he developed diarrhoea on 16 June while fishing on Lake Edward. Subsequently he returned to the shore during the early hours on 17 June, and reportedly defecated “near” water-collection site X. He received treatment at Bwera Hospital (the regional referral hospital) on 17 June, where he was laboratory-confirmed to have cholera.

### Environmental and laboratory investigation findings

Each of the five settlements in Katwe Village had their own water-collection sites. Because there are dangerous lake animals (e.g., Nile crocodiles and hippopotamuses) in Lake Edward, residents of the village used rocks and a fence to surround water-collection points to protect water-collecting villagers, especially children and those collecting water during darkness, from potential attacks of lake animals.

**Fig 3:**
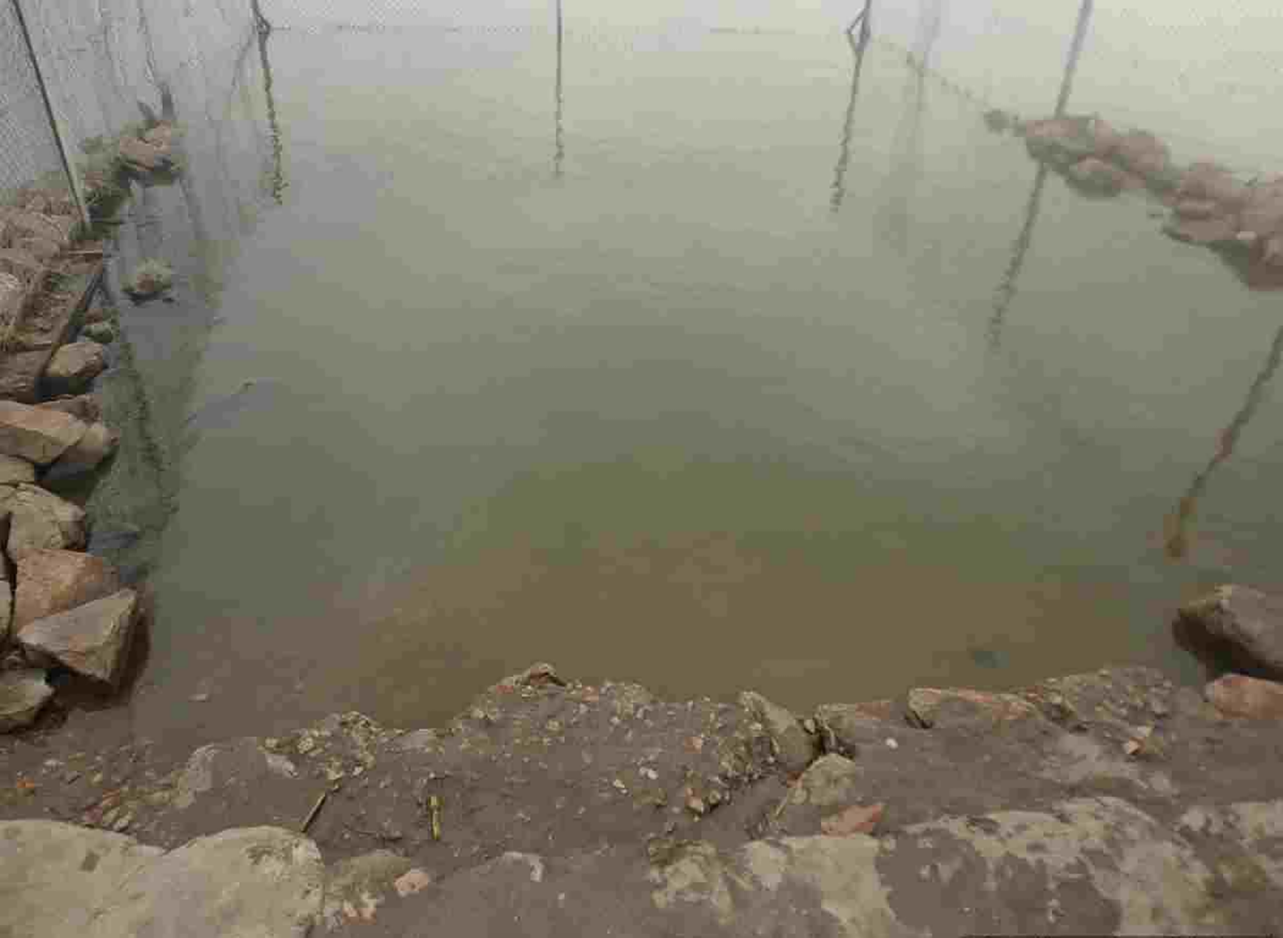
Water-collection site X on the shore of Lake Edward, Katwe Village, south-western Uganda. Water collected inside this fenced area was implicated for a cholera outbreak during June–July 2015.

Water-collection site X was located on the shore of Lake Edward, which served the Kyarukara settlement zone. Rocks and fences surrounding the collection site potentially made the lake water inside stagnant, preventing contaminants from being diluted quickly (Fig 3). We noted during our environmental investigation that not all villagers in the Kyarukara settlement zone collected water inside the fenced area of water-collection site X; some collected water outside of the fenced area. The environmental Investigation showed water collected point implicated during the cholera outbreak (Fig 3).

Katwe Village used to have a tap-water system. However, the system broke down eight months prior to this outbreak. The village also had a protected spring, which provided cleaner water. The spring was about three kilometres away from the village centre, up on a hill. After climbing up the hill, a water collector often would have to wait in the line for hours to get a jerry-can of water. A commercial group collected and sold the spring water to the villagers at 1000 Uganda shillings (about US$1.5) per 100 litres, which is approximately the amount of water used by an average household in the village. This price was not affordable by most of the villagers; therefore they used the free lake water for drinking and other household use.

Interviews of community leaders also revealed that during the initial few days after the occurrence of the outbreak, some case-patients’ family members washed soiled clothes nearwater-collection site X. After health education was conducted, this practice stopped, as observed by the outbreak investigation team during the investigation.

Laboratory investigations found that 19 of the 27 samples collected from case-patients were positive for *V*. *cholerea* by RDT while eight were positive for *V*. *cholerea* O1 (serotype Inaba) by stool culture.

## Discussion

Our investigation demonstrated that the cholera outbreak in Katwe Village, south-western Uganda during June–July 2015 was a point-source outbreak caused by drinking contaminated water collected from the inside of a water-collection site on the shore of Lake Edward.

Globally, waterborne disease outbreaks often occur in countries where water and sanitation conditions are poor [17–21]. Rural Sub-Saharan Africa has had one of the highest population growth rates globally in recent years, yet access to improved water source and sanitation facilities has not changed much during the same time period [22]. Consequently, outbreaks of waterborne diseases (including cholera, typhoid fever, bacterial dysentery, and hepatitis E) often occur, endangering the lives and wellbeing of millions of people not just in Sub-Saharan Africa but in the entire global community as a whole [23]. Of these waterborne diseases, cholera has had an especially large impact on Africa countries since the seventh pandemic reached the continent during the 1970s, with a high reported incidence and case-fatality rates [23–26].

In Uganda, an investigation of a previous cholera outbreak that occurred among the northern country’s semi-nomadic pastoralists revealed that the outbreak was also caused by drinking untreated water [27]. During other recent cholera outbreaks, drinking water contamination was often implicated or assumed, although rigorous epidemiologic investigations rarely have been conducted [8, 28].

History of public health has demonstrated time and again that early identification, investigation and response are the key for prompt control of communicable disease outbreaks. This outbreak started on 16 June and was reported on 20 June. We started our investigation on 22 June. During the outbreak investigation, we recommended to the village administrators to provide water chlorination tablets to the villagers, issue water-boiling advisory to the villagers, and rigorously disinfect all patients’ faeces. With the assistance of the investigation team, the village administrators implemented all of these recommendations. After 22 June, eight additional cases occurred sporadically, likely due to the transmission of *V*. *cholerae* from the initial patients, possibly through washing of their soiled clothes in the water-collection site during the initial few days of the outbreak, as reported by the village leaders. The outbreak completely stopped after 1 July 2015 after control measures were aggressively implemented. In comparison, elsewhere in Kasese District, an outbreak started in another community in late February 2015 but was not responded to until May 2015. That outbreak spread to many communities and lasted several months [10]. While it is impossible to prove that the prompt investigation and aggressive response during our investigation stopped this outbreak early, our findings serve as a reminder of the importance of early detection, prompt detection and rapid response when an outbreak occurs.

## Conclusion

In conclusion, this cholera outbreak was caused by drinking contaminated water from a lakeshore water-collection site. The primary case-person’s faeces briefly contaminated the water inside the fenced water-collection site. The water was made stagnant by the rocks and the fence surrounding the site, which might have facilitated this outbreak.

## Supporting information

S1 Case Control (ZIP)
